# Role of Histopathology in Determining the Cause of Death in Medicolegal Autopsies

**DOI:** 10.7759/cureus.81055

**Published:** 2025-03-23

**Authors:** Bhaskar Mukherjee, Amarantha Donna Ropmay, Daunipaia Slong, Prabal Das, Amar J Patowary, Yookarin Khonglah, Lokesh Ravi V Naidu, Rohan Das

**Affiliations:** 1 Forensic Medicine, North Eastern Indira Gandhi Regional Institute of Health & Medical Sciences, Shillong, IND; 2 Pathology, North Eastern Indira Gandhi Regional Institute of Health & Medical Sciences, Shillong, IND

**Keywords:** cause of death, gross autopsy, histopathology, medico-legal autopsy, north east india

## Abstract

Background

A medicolegal or forensic autopsy is performed on the instructions of the legal authority responsible for the investigation of sudden, suspicious, obscure, unnatural, litigious, or criminal deaths. Although the purpose and procedure of a medicolegal autopsy differ from those of a pathological autopsy, at times, they overlap. Usually, histopathological examination (HPE) is required in sudden deaths, where the cause of the death is not known or not apparent at gross autopsy.

Purpose

This research has been conducted for a tenure of one and a half years, from October 2022 to April 2024, analyzing 87 autopsy cases in a tertiary care center in Northeast India to determine the role of histopathological examination in medicolegal autopsies.

Method

Data collection involved detailed histopathological examinations and a structured pro forma to ensure comprehensive documentation of the findings. The study's independent variables include age, sex, and time since death, while outcome variables focus on histopathological findings and the determined cause of death. Data was analyzed using IBM SPSS Statistics for Windows, Version 28.0.1.1 (Released 2022; IBM Corp., Armonk, New York, United States). A p-value of less than 0.05 at a 95% confidence interval (CI) was considered statistically significant.

Results

An analysis of the provisional cause of death determined through gross autopsy and the final opinion on the cause of death established by histopathology revealed a statistically significant association between the two. However, there is less than a chance of agreement among them, as indicated by a Kappa statistic of -0.497. Among the 47 cases where a provisional cause of death was determined through gross autopsy, the final cause of death was not concluded based on histopathological examination (HPE) in 45 cases (95.7%). Additionally, in the 23 cases where the provisional cause of death was pending until the HPE report was received, the final opinion on the cause of death was determined based on HPE in 21 cases (91.30%). Analyzing the final opinion on the cause of death and the opinions based on HPE, it was found that their relationship is statistically significant as the p-value was calculated to be <0.05.

Conclusion

In medicolegal autopsies, histopathology is essential for determining the cause of death when there are no visible external injuries or when the cause of death is unclear at the time of the gross autopsy. When the preliminary cause of death is known at the time of the gross autopsy, such as in cases of fatal injuries, the role of histopathological examination is restricted.

## Introduction

The legal authority in charge of looking into abrupt, strange, enigmatic, unnatural, litigious, or criminal deaths orders the medicolegal or forensic autopsy [1]. General pathologists conduct clinical postmortems or autopsies to identify malignant illnesses, infectious diseases, and subclinical diseases and to educate the family about inheritable diseases. Although histological analysis is typically performed during medicolegal autopsies, it is not as frequently done as during clinical autopsies, which is a proven method of education and quality assurance [2-4]. The division of pathology known as histopathology deals with tissue, or "histo." The duty of a histopathologist is to examine tissue and cells and determine whether they exhibit any unusual characteristics. It provides insights into the cause of death and aids in the resolution of doubts regarding the cause of death by describing the microscopic investigation of numerous pathophysiological abnormalities at the cellular level [5].

Whether or not all autopsy cases, irrespective of cause, manner, and mode of death, should receive histological analysis has been debated for a very long time. Regular histological testing demonstrates thoroughness, shows that the autopsy findings may be reviewed, and raises the level of trust that can be placed in the findings and conclusions. The pathologists perform histopathological tests to confirm their macroscopic findings and to substantiate them [6]. Histology is mostly employed in the field of forensic pathology to recognize some illnesses, such as myocarditis, amniotic fluid embolisms, and fat embolisms, which can only be diagnosed under a microscope. The only way to diagnose some disorders, which have specific histological traits, is by autopsy histopathology. Brain, lungs, heart, liver, adrenal glands, and kidneys with normal external appearance may have important histopathological findings [7,8]. Although histological injury timing has drawn a lot of interest, its practical applications are not as ubiquitous as one might anticipate. Studies have shown that in situations when the cause of death is readily apparent at the time of autopsy, such as in fire, immersion, suspension, and traffic-related deaths, microscopic inspection does not reveal further information concerning infectious or inheritable disease processes [7,9-11]. In addition to other systemic responses to injury, microscopic analysis can be utilized to support the presence of pulmonary embolism, ischemia, and sepsis [7].

India is a developing nation. It is always important to keep in mind that resource constraints are more important than resource optimization. No different from other medical professionals, forensic pathologists must choose which cases during autopsies require histopathological analysis and which ones do not, serving the purpose of using resources wisely. This study, which was conducted in a tertiary care center in Northeast India over a one-and-a-half-year period, aimed to do an in-depth analysis of the histopathological findings of medicolegal autopsies to determine the rationality of performing routine histopathological examination (HPE) in all the medicolegal autopsies.

## Materials and methods

Study design 

It was a cross-sectional analytical prospective study conducted in the Department of Forensic Medicine of North Eastern Indira Gandhi Regional Institute of Health & Medical Sciences, a tertiary care hospital in Northeast India (Shillong, Meghalaya) from October 2022 to April 2024 after getting approval from the Institutional Ethics Committee (IEC) (NEIGR/IEC/M7/T7/2022, dated October 22, 2022). 

Primary objective

To assess the role of the histopathological examination (HPE) in determining the cause of death in medicolegal autopsy cases.

Research question

“What is the precise role of histopathological examination in determining the cause of death in the cases brought for medicolegal autopsies?”

Research hypothesis

“There is a definite role of histopathological examination in medicolegal autopsies to find out the cause of death.”

Sampling

A total of 87 samples were collected, using a convenient sampling method, from the cases brought for medicolegal autopsies to the hospital mortuary for one and a half years after obtaining informed consent from the next of kin of the deceased. Cases showing advanced decomposition and non-viable fetuses and cases where consent was not given by the relatives were excluded from the study. The cases were subjected to thorough dissection where the brain, heart, lungs, kidney, and liver were removed as per techniques suggested by Ludwig [12]. The removed organs were fixed in a 10% formalin solution and were further subjected to detailed HPE using hematoxylin & eosin stain to find out the cause of death. 

Data collection

Data was collected during the medicolegal autopsy as per the prescribed pro forma and after informed consent and from reports of detailed histopathological examination. Independent variables were age, sex of the study cases, and time since death, and outcome variables were histopathological findings and cause of death.

Data analysis

Data was analyzed using IBM SPSS Statistics for Windows, Version 28.0.1.1 (Released 2022; IBM Corp., Armonk, New York, United States). Categorical variables were presented as frequency and percentages. The chi-square test was used to determine the association between the categorical variables. A p-value of less than 0.05 was considered statistically significant.

## Results

Analysis showed a fair association between the provisional cause of death on gross autopsy and the presence or absence of visible external injury (using Fisher’s exact test) (Table 1). The Kappa value was 0.385. It was also found that out of the 61 cases where external injury was present, in 70.5% of cases, the provisional cause of death could be established based on only gross autopsy.

**Table 1:** Frequencies of the cases with the presence or absence of external injury and the provisional cause of death based on gross autopsy X2 (3, N = 87) = 26.890; p = 0.000 (statistically significant); Kappa value = 0.385 (fair agreement); HPE: histopathological examination; FSL: forensic science laboratory

Provisional cause of death based on gross autopsy		External injury		Total
		Present	Absent	
Established	Frequency	43	4	47
	% within external injury	70.5%	15.4%	54.0%
Kept pending till receipt of HPE report	Frequency	8	15	23
	% within external injury	13.1%	57.7%	26.4%
Kept pending till receipt of FSL report	Frequency	0	1	1
	% within external injury	0.0%	3.8%	1.1%
Kept pending till receipt of both HPE and FSL report	Frequency	10	6	16
	% within external injury	16.4%	23.1%	18.4%
Total	Frequency	61	26	87
	% within external injury	100.0%	100.0%	100.0%

Analyzing the provisional cause of death on gross autopsy and the presence or absence of a history of natural disease, it was found that statistically there is no significant relationship between those (Table 2). It was found that out of 12 cases in which there was a positive history of any natural disease, the provisional cause of death could be established in 41.7% of the cases on gross autopsy; it was kept pending till the arrival of the HPE report and the arrival of both the HPE and forensic science laboratory (FSL) report, respectively, in 41.7% and 16.7% of the cases.

**Table 2:** Frequencies of the cases with a known or unknown history of natural disease and the provisional cause of death based on gross autopsy X2 (3, N = 87) = 2.292; p = 0.553 (statistically insignificant); HPE: histopathological examination; FSL: forensic science laboratory

Provisional cause of death based on gross autopsy		History of natural disease		Total
		Known	Unknown	
Established	Frequency	5	42	47
	% within history of natural disease	41.7%	56.0%	54.0%
Kept pending till receipt of HPE report	Frequency	5	18	23
	% within history of natural disease	41.7%	24.0%	26.4%
Kept pending till receipt of FSL report	Frequency	0	1	1
	% within history of natural disease	0.0%	1.3%	1.1%
Kept pending till receipt of both HPE and FSL report	Frequency	2	14	16
	% within history of natural disease	16.7%	18.7%	18.4%
Total	Frequency	12	75	87
	% within history of natural disease	100.0%	100.0%	100.0%

Analyzing the final opinion on the cause of death and the final opinion based on HPE, a statistically significant relationship was found among them as the p-value was calculated to be <0.05 (Table 3), and also there is slight agreement among them as the value of the Kappa statistics was calculated to be 0.004. Out of three cases of neoplasm, in all three cases (100%), a final opinion was given based on histopathological examination. Similarly, in all the cases of multiorgan failure, chronic ischemic heart disease, chronic interstitial pulmonary disease, acute respiratory distress syndrome, chest injury, pulmonary tuberculosis, aspiration pneumonia, abdominal injury, and pneumonitis, the final opinion on the cause of death was given based on histopathological examination.

**Table 3:** Frequencies of the cases in which the final opinion was or was not based on histopathology and the final opinion on the cause of death X2 (17, N = 87) = 61.009; p = 0.000 (statistically significant)

Final opinion on the cause of death		Final opinion based on histopathology		Total
		Yes	No	
Acute myocardial infarction	Frequency	3	0	3
	% within final opinion on cause of death	100.0%	0.0%	100.0%
Cerebrovascular accident	Frequency	3	1	4
	% within final opinion on cause of death	75.0%	25.0%	100.0%
Head injury	Frequency	1	21	22
	% within final opinion on cause of death	4.5%	95.5%	100.0%
Final opinion is pending	Frequency	1	5	6
	% within final opinion on cause of death	16.7%	83.3%	100.0%
Poisoning	Frequency	1	4	5
	% within final opinion on cause of death	20.0%	80.0%	100.0%
Neoplasm	Frequency	3	0	3
	% within final opinion on cause of death	100.0%	0.0%	100.0%
Multiorgan failure	Frequency	1	0	1
	% within final opinion on cause of death	100.0%	0.0%	100.0%
Antemortem drowning	Frequency	0	3	3
	% within final opinion on cause of death	0.0%	100.0%	100.0%
Antemortem hanging	Frequency	2	12	14
	% within final opinion on cause of death	14.3%	85.7%	100.0%
Chronic ischemic heart disease	Frequency	7	0	7
	% within final opinion on cause of death	100.0%	0.0%	100.0%
Hemorrhagic shock	Frequency	1	10	11
	% within final opinion on cause of death	9.1%	90.9%	100.0%
Chronic interstitial pulmonary disease	Frequency	1	0	1
	% within final opinion on cause of death	100.0%	0.0%	100.0%
Acute respiratory distress syndrome	Frequency	1	0	1
	% within final opinion on cause of death	100.0%	0.0%	100.0%
Chest injury	Frequency	2	0	2
	% within final opinion on cause of death	100.0%	0.0%	100.0%
Pulmonary tuberculosis	Frequency	1	0	1
	% within final opinion on cause of death	100.0%	0.0%	100.0%
Aspiration pneumonia	Frequency	1	0	1
	% within final opinion on cause of death	100.0%	0.0%	100.0%
Abdomen injury	Frequency	1	0	1
	% within final opinion on cause of death	100.0%	0.0%	100.0%
Pneumonitis	Frequency	1	0	1
	% within final opinion on cause of death	100.0%	0.0%	100.0%
Total	Frequency	31	56	87
	% within final opinion on cause of death	35.6%	64.4%	100.0%

Analyzing the provisional cause of death based on gross autopsy and final opinion on the cause of death based on histopathology, it was found that the association among them is statistically significant, but there is less than chance agreement among them, as the Kappa statistics show the value to be -0.497. It was found that out of 47 cases, in which the provisional cause of death based on gross autopsy could be established, in 45 cases (95.7%), the final opinion on the cause of death was not given based on HPE. It was also found that out of 23 cases in which the provisional cause of death was kept pending till the arrival of the HPE report, in 21 cases (91.30%) the final opinion on the cause of death was given based on HPE (Table 4).

**Table 4:** Frequencies of the cases in which the final opinion was or was not based on histopathology and the provisional cause of death based on gross autopsy Fisher’s exact test = 58.132 (p-value <0.001); Kappa = -0.497 (no agreement); HPE: histopathological examination; FSL: forensic science laboratory

Provisional cause of death based on gross autopsy		Final opinion based on histopathology		Total	p-value
		Yes	No		
Established	Count	2	45	47	<0.001
	% within provisional cause of death based on gross autopsy	4.3%	95.7%	100.0%	
Kept pending till receipt of HPE report	Count	21	2	23	
	% within provisional cause of death based on gross autopsy	91.3%	8.7%	100.0%	
Kept pending till receipt of FSL report	Count	0	1	1	
	% within provisional cause of death based on gross autopsy	0.0%	100.0%	100.0%	
Kept pending till receipt of both HPE and FSL report	Count	8	8	16	
	% within provisional cause of death based on gross autopsy	50.0%	50.0%	100.0%	
Total	Count	31	56	87	
	% within provisional cause of death based on gross autopsy	35.6%	64.4%	100.0%	

## Discussion

According to Jhajj et al.'s study, the most common diagnosis in the cardiovascular system was cardiomegaly (10.4%), which was followed by giant cell myocarditis (0.8%), atherosclerosis (3.2%), myocardial infarction (8.8%), and thrombus (0.8%) [13]. In the current study, the most common histopathological finding of the heart was healthy myocardium (80.46%), followed by hypertrophy (12.64%). It was also found that the final opinion on the cause of death was given as acute myocardial infarction in three cases (Table 3), whereas the opinion was given only after histopathological examination in all three (100%) cases. Out of seven cases where the final opinion about the cause of death was given as chronic ischemic heart disease, the opinion was made only after histopathological examination in all seven cases (100%) (Table 3).

Pathak and Mangal, in their study, found congestion was the commonest histopathological finding in 52 cases (57.7%), followed by edema in 30 cases (33.3%), pneumonia in 22 cases (24.4%), and tuberculosis in 12 cases (13.3%) in the respiratory system [14]. In the current study, the commonest histopathological finding of the lung was normal histology in both the right and left lungs (43 out of 87) (49.43%), followed by congestion in 19.54% and 20.69% of cases, respectively, in the left and right lungs. The final opinion on the cause of death was given as chronic interstitial pulmonary disease, acute respiratory distress syndrome, pulmonary tuberculosis, and aspiration pneumonia in one case each, but all the cases (Table 3) were diagnosed only after the histopathological examination.

Gahine et al. reported that in the central nervous system, the most prevalent histological result (23.6%) was cerebral edema, and that involvement of the central nervous system was the sixth most common cause of death, accounting for 31.5% of cases [15]. However, in the current study, normal histology was the most common finding in the brain, which was found in 68 cases (78.16%). The brain was found congested in 73 cases (83.91%) on gross examination, whereas in nine cases (1.34%) it was found on microscopic examination. It was also found that, out of four cases where the final opinion was given as cerebrovascular accident (Table 3), the opinion was given after histopathological examination in three cases (75%), and only in one case (25%) was it not given on the basis of histopathological examination.

Pathak and Mangal, in their study, found congestion of the liver in 44 cases (48.8%), fatty changes in eight (8.8%) cases, and cirrhotic changes in two (2.2%) cases on postmortem HPE in the hepatobiliary cause [14]. In the current study, on HPE, the tissue of the liver was healthy in 46 cases (52.87%), which was the most common finding, and cirrhotic changes were found in five cases (5.75%). It was also found that when the size of the liver was grossly normal, the commonest finding on histopathological examination was healthy histology.

In their investigation, Pathak and Mangal observed that congestion was the most frequent finding in the kidney on HPE. This condition was found in 42 instances (46.6%), and coagulative necrosis in 38 cases (42.2%) [14]. In the current study, the most common histopathological finding was healthy histology (60.92%) in both the right and left kidneys, followed by congestion, which was found in 12.64% of the cases in the left kidney and in 13.79% of the cases in the right kidney.

In the current study, it has been statistically proven that histopathology plays a significant role in finding out the cause of death in cases where there is a history of any natural disease prior to death, and it has also been described that the role of histopathology is limited when the cause of death is evident at the time of gross autopsy. It has also been described that histopathological examination is vital to diagnose neoplastic cases (Table 3) and also the conditions that are chronic in nature but were not diagnosed during the alive state. It has also been found that the role of routine histopathological examination is limited when there is the presence of external injury at the time of gross autopsy.

Limitations

Although corpses with external signs of advanced decomposition have been excluded from the study, there were a few cases where, during the microscopic examination, autolytic changes have been encountered. Another limitation was the role of HPE in finding out the cause of death in gunshot injuries could not be elicited, as no such case was received during the study period for medicolegal autopsy.

Recommendations

Studies with a greater number of samples and including more organs, as well as studies using special stains, are recommended for the future.

## Conclusions

In medicolegal autopsies, histopathology is essential for determining the cause of death when there are no visible external injuries or when the cause of death is unclear during gross autopsy. Histopathology is an important modality in coming to a postmortem diagnosis of ailments that were not diagnosed during life. When the preliminary cause of death is known at the time of the gross autopsy, such as in cases of fatal injuries, the role of histopathological examination is restricted.
